# Knee Angle Estimation with Dynamic Calibration Using Inertial Measurement Units for Running

**DOI:** 10.3390/s24020695

**Published:** 2024-01-22

**Authors:** Matthew B. Rhudy, Joseph M. Mahoney, Allison R. Altman-Singles

**Affiliations:** 1 Mechanical Engineering, The Pennsylvania State University, Berks College, Reading, PA 19610, USA; 2 Mechanical Engineering, Alvernia University, Reading, PA 19607, USA; joseph.m.mahoney@gmail.com; 3 Kinesiology, The Pennsylvania State University, Berks College, Reading, PA 19610, USA; ara5093@psu.edu

**Keywords:** inertial measurement units, gait analysis, kinematic constraints, Kalman filtering

## Abstract

The knee flexion angle is an important measurement for studies of the human gait. Running is a common activity with a high risk of knee injury. Studying the running gait in realistic situations is challenging because accurate joint angle measurements typically come from optical motion-capture systems constrained to laboratory settings. This study considers the use of shank and thigh inertial sensors within three different filtering algorithms to estimate the knee flexion angle for running without requiring sensor-to-segment mounting assumptions, body measurements, specific calibration poses, or magnetometers. The objective of this study is to determine the knee flexion angle within running applications using accelerometer and gyroscope information only. Data were collected for a single test participant (21-year-old female) at four different treadmill speeds and used to validate the estimation results for three filter variations with respect to a Vicon optical motion-capture system. The knee flexion angle filtering algorithms resulted in root-mean-square errors of approximately three degrees. The results of this study indicate estimation results that are within acceptable limits of five degrees for clinical gait analysis. Specifically, a complementary filter approach is effective for knee flexion angle estimation in running applications.

## 1. Introduction

Running is an important aspect of many people’s lives through various sports, leisure, and fitness activities [1]. Despite its popularity, running is often associated with a high risk of injury [2,3,4]. The knee is a common site for overuse injuries [5,6,7]. Knee injuries have been associated with both knee kinematics and kinetics in previous studies [8,9]. In addition, knee flexion angles are important data when assessing various aspects of gaits in different research applications, such as assessing the joint angle symmetry during fatigue [10], comparing male and female runners [11], and performing gait analysis for people with multiple sclerosis [12], among others. Knee flexion is associated with higher strain in the knee ligaments, which can result in ligament strains and tears when paired with other dynamic movements. Knee flexion is also important for optimizing performance and making comparisons between different demographic groups. Because running is a common physical activity that presents a risk of knee injury, research regarding knee angle estimation during running is necessary to clearly understand gait kinematics.

Various measurement systems have been used for gait analysis [13] to determine knee flexion angles. Optical motion capture is a popular way to identify joint angles owing to its high accuracy [14]. This technology, however, is cost limiting for some researchers. Additionally, the application becomes limited to relatively small testing areas owing to the physical constraints of fixed motion-capture camera systems. The process for applying markers is also time consuming. This is especially restrictive when considering studies of gait during running because much of running is done outdoors. Outdoor activity is often encouraged for individuals because of the overall health benefits of the time spent outdoors [15,16]. Owing to these significant limiting factors of optical motion-capture systems, portable technology for identifying joint angles is desirable. That is, optical motion-capture systems are not practical for analyzing gaits during outdoor running applications, such as trail running and running during outdoor sports.

Inertial measurement units (IMUs) have become a popular means for identifying human kinematic information in various applications owing to their reasonable availability, cost, and portability. The wearable nature of these sensors makes them practical for use in different environments outside of a laboratory. Because these sensors are wearable, they can be used in outdoor running applications as a means for estimating knee angles. IMUs have been used in multiple studies to estimate the knee flexion angles for applications, such as walking [17,18,19,20], walking and running [21,22,23], squats [21,24], lunges [25], and joint rehabilitation movements [26].

IMUs typically contain three-axis accelerometers, three-axis rate gyroscopes, and, sometimes, three-axis magnetometers. Although the details of the actual implementation vary, the general principles of IMU knee-flexion-angle estimation research involve the fusion of different combinations of IMU sensor information through filtering techniques, such as the complementary filter (CF) [17], Kalman filter (KF) [22], extended Kalman filter (EKF) [20,21,22,26], and unscented Kalman filter (UKF) [18,24], or smoothers, such as the Rauch–Tung–Striebel smoother (RTSS) [25]. Some researchers consider the use of accelerometers, rate gyroscopes, and magnetometers [18,24]; however, more commonly, accelerometers and rate gyroscopes are implemented without including magnetometer measurements [17,19,20,21,22,25,26]. Magnetometer measurements are often omitted from joint estimation algorithms owing to their susceptibility to interference from local magnetic fields [20,25].

Other researchers have considered data-driven approaches, such as machine learning, for knee-flexion-angle estimation [23,27,28]. For example, Gholami et al. consider foot-mounted accelerometer data coupled with machine learning to identify lower limb kinematics [23]. As noted in Gholami et al. [23], however, there are significant limitations with data-driven approaches for joint angle estimation applications. Specifically, it is difficult to generalize the results of this type of study for a general population owing to the possibility of overfitting to the sample data. Additionally, a large and diverse dataset must be collected to train and validate these types of approaches. Because of this, data-driven approaches are not considered as a part of this article.

One of the challenges associated with the use of IMU data for joint angle estimation is the alignment of the sensor frames to the relevant body segments. There are different strategies for handling this issue, each with advantages and disadvantages. As outlined by Hindle et al. [24], the commonly implemented strategies for sensor-to-segment alignment can be categorized into manual alignment [29], static pose estimation [18,19,22,30], functional calibration [31,32,33], deep learning [34], or some combination of these approaches [21]. The use of static poses is common, according to the literature, involving subjects holding still from 5 to 10 s in a specified standing pose [18,19,21,22] or seated pose [19]. For these static data, accelerometers are utilized to detect the gravity vector because all the accelerations measured using accelerometers are due to only gravity when there is no motion [19,21]. Alternatively, some researchers use estimated orientations of the individual IMUs from a filtering algorithm to perform the static alignment [18,24]. The other commonly considered approach for sensor-to-segment alignment is functional calibration, which requires the subjects to execute specific calibration movements prior to the testing. These movements are selected to excite different modes of motion in the anatomical planes, thus increasing the observability of the body segments within the IMU data. Knee flexion and extension movements were used either actively or passively by a physical therapist in Cutti et al. [32] and Ferrari et al. [33]. Favre et al. consider a combination of multiple active and passive movements for calibration purposes [31].

In addition to these techniques, some research efforts have investigated dynamic calibration procedures that do not require any specific static poses or calibration movements prior to the testing. This idea, originally proposed by Seel et al. [17,35], was later utilized with some modifications by Versteyhe et al. [25] and Vasquez et al. [20]. In each of these studies, some notable advantages have been identified as follows [17]:

1. Sensor-to-segment mounting assumptions are avoided;
2. No measurements of body segment distances are manually determined;
3. No predefined postures or movements are required for the calibration;
4. Magnetometers are not required.

Another study by McGrath et al. proposes a similar approach but using a different mathematical technique: principal component analysis (PCA) [36]. Carcreff et al. explored PCA for sagittal plane identification using foot-mounted IMUs [37].

The principles of the dynamic calibration method by Seel et al. [17] have so far been implemented for level walking [17,20], stair descent [20], sit-to-stand-to-sit [20], and lunges [25]. These movements involve relatively low accelerations and low impacts when compared to running. Preliminary implementations of the method by Seel et al. [17] for running data resulted in somewhat poor estimates of knee flexion angles, thus motivating a need for improving and refining this technique for the specific application of running.

IMU data have been applied to running tasks in multiple previous studies. For example, accelerometer data were used to compare male and female running patterns using machine learning in Clermont et al. [38]. Wundersitz et al. used trunk-mounted accelerometer data to compare peak accelerations experienced during walking, jogging, and running relative to a motion-capture system [39]. A tibial accelerometer was used in [40] to estimate the ground reaction force during running. IMUs have been used to identify initial contact and terminal contact gait events in Kiernan et al. [41] and Yang et al. [42]. Potter et al. conducted a study of foot IMUs for estimating running gait parameters [43]. Building upon the insight of these studies, this study incorporates the use of IMU data to identify key aspects of the running gait and leverages this information to capture the knee angle kinematics more effectively.

The objective of this study is to expand upon the work of Seel et al. [17,35] to make additional novel contributions. The following items outline the key differences in this study:

1. The methods of Seel et al. [17] are adapted to work well, specifically for the application of running, which involves fast movements with high accelerations. The methodology of Seel et al. [17] does not perform well when directly applied to running data;
2. Simple accelerometer peak detection is used to parse the data into gait cycles. This gait cycle information is then leveraged to separate the IMU data into regions of data corresponding to different aspects of the gait, such as the stance phase, swing phase, foot strike, and float. Based on this information, the dynamic alignment of the sensors is performed strategically, using the reliable portions of the dataset. This improves the necessary sensor alignment, thus allowing for more accurate knee flexion angle estimates;
3. Using the newly aligned sensor data, three different filtering algorithms are developed for the application of knee flexion angle estimation during running. These algorithms utilize rate gyroscope and accelerometer measurements from one shank-mounted and one thigh-mounted IMU. Each of these algorithms is compared with respect to accurate reference data from a motion-capture system;
4. The identified foot strikes are also used to identify the portions of the accelerometer data that correspond to the impact rather than the dynamic movement. These portions of the dataset are strategically omitted from the measurement update portion of the proposed filtering algorithms, thus mitigating the erroneous effects of the impact due to foot strikes.

## 2. Materials and Methods

### 2.1. Experimental Setup and Data Acquisition

#### 2.1.1. Inertial Measurement Units

**Figure 1:**
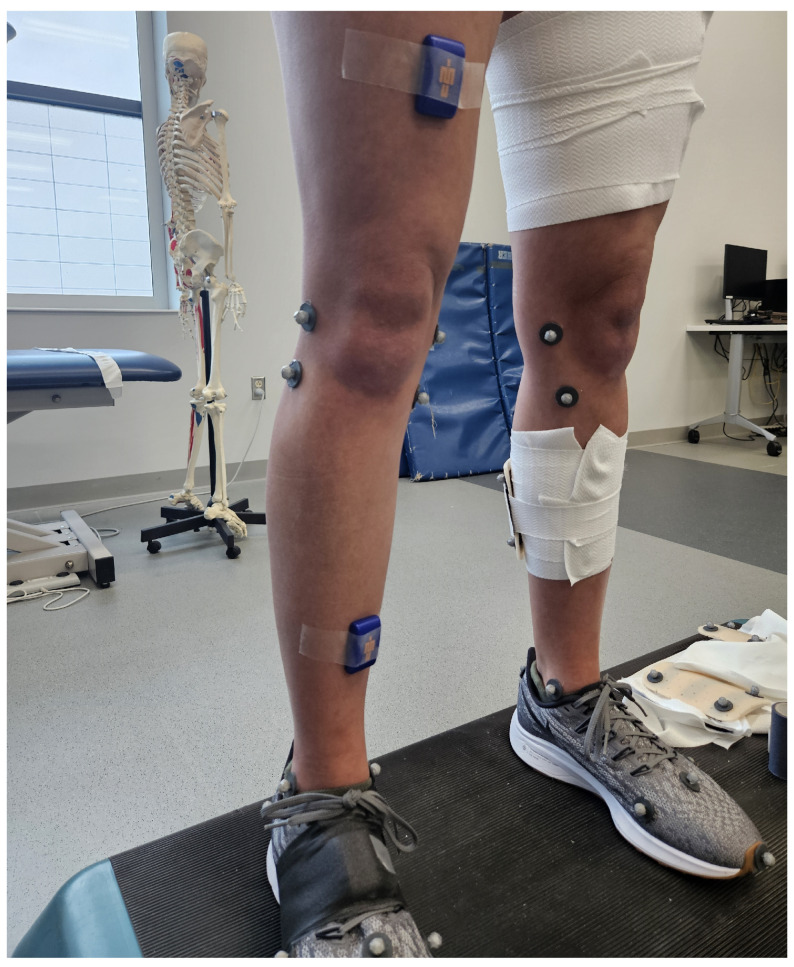
Image of the IMU mounting on the participant.

This study utilized two Blue Trident (Vicon, Centennial, CO, USA) inertial measurement units (IMUs) fixed to the anterior distal aspect of the thigh and the anteromedial distal aspect of the tibia of the dominant leg. Each was taped to the skin and wrapped firmly to the corresponding segment. A picture of the IMU mounting locations is provided in Figure 1 prior to being wrapped. The considered IMUs feature accelerometer measurements for a range of ±16 g with 16-bit resolution and gyroscope measurements for a range of ±2000 deg/s with 16-bit resolution. These IMUs also contain magnetometers; however, these measurements were not used for this study.

#### 2.1.2. Motion-Capture System

In addition to the IMUs tracking the thigh and shank segments, 23 retroreflective markers were taped to the dominant limb of the participant. Note that the same leg was used for the motion capture and IMU sensor mounting for this study. A twelve-camera combined Vicon Bonita and Vero (Vicon, Centennial, CO, USA) motion-capture system was used to collect optical marker data on the thigh and shank of the lower extremity. All the data were collected using Vicon Nexus 2.15 (Vicon, Centennial, CO, USA) at 200 Hz and synchronized with the IMU data collected at 400 Hz. A standing calibration of the participant was collected, and the anatomical markers were removed, leaving only the IMUs and tracking marker clusters on the thigh and shank.

The raw marker trajectories were low-pass filtered using a zero-lag 4th-order Butterworth filter with a 6 Hz cutoff frequency. The knee and thigh segments were identified, and the 3D joint angles were calculated using the optical markers in Visual 3D (C-Motion Inc., Germantown, MD, USA).

### 2.2. Knee Flexion Angle Estimation Using Shank- and Thigh-Mounted IMUs

**Figure 2:**
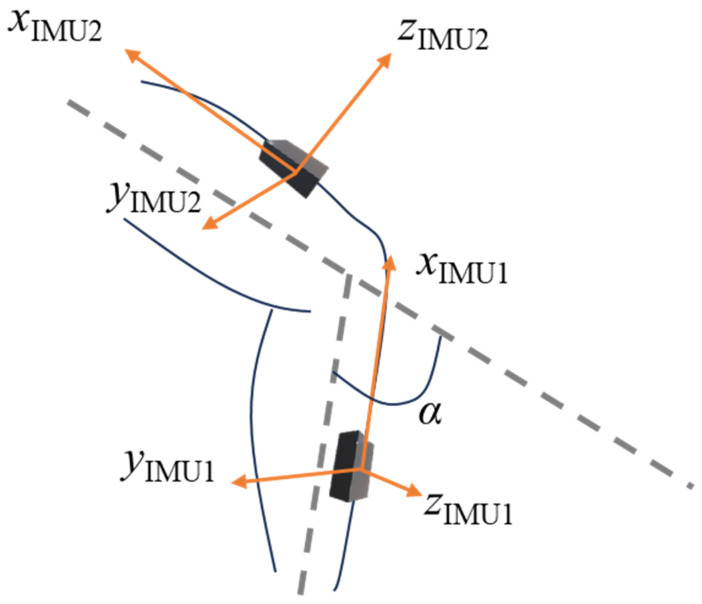
Diagram of IMU mounting locations, coordinate systems, and knee flexion angle, *α*.

This study considers the estimation of knee flexion angles using two IMUs. One IMU is mounted on the shank, which is indicated as IMU1, while the other is mounted on the thigh, which is indicated as IMU2. A diagram illustrating the mounting location and IMU axes and the knee flexion angle, *α*, is shown in Figure 2.

#### 2.2.1. IMU-Based Joint Angle Measurement for Gait Analysis [17]

Consider the shank and thigh IMUs, as shown in Figure 2, which provide triaxial accelerations *a*_1_(*t*) and *a*_2_(*t*) and triaxial angular velocities *g*_1_(*t*) and *g*_2_(*t*) for a sampling period, Δ*t*, where the subscripts 1 and 2 indicate the shank and thigh, respectively. The angular accelerations are calculated using a third-order central differencing approximation as follows:

(1) $${\dot{g}}_{i}\left(t\right)=\frac{g_{i}\left(t-2\Delta t\right)-8g_{i}\left(t-\Delta t\right)+8g_{i}\left(t+\Delta t\right)-g_{i}\left(t+2\Delta t\right)}{12\Delta t},\;\;\;i=1,2$$

An optimization procedure is used to determine the 3D unit vectors, *j*_1_ and *j*_2_, which correspond to the knee flexion axis in the local coordinates of the shank and thigh sensors, respectively. This is accomplished by minimizing the cost function as follows:

(2) $$\begin{array}{l} \Psi \left(\phi_{1},\phi_{2},\theta_{1},\theta_{2}\right)=\sum_{k=1}^{N}{\left[{\left\|g_{1}\left(t_{k}\right)\times j_{1}\right\|}_{2}-{\left\|g_{2}\left(t_{k}\right)\times j_{2}\right\|}_{2}\right]}^{2}\\ \begin{array}{cc} j_{1}=\left[\begin{array}{c} \cos\phi_{1}\cos\theta_{1}\\ \cos\phi_{1}\sin\theta_{1}\\ \sin\phi_{1} \end{array}\right], & j_{2}=\left[\begin{array}{c} \cos\phi_{2}\cos\theta_{2}\\ \cos\phi_{2}\sin\theta_{2}\\ \sin\phi_{2} \end{array}\right] \end{array} \end{array}$$

where ${\left\|\cdot \right\|}_{2}$ denotes the *L*_2_-norm, (*ϕ*_1_, *θ*_1_) and (*ϕ*_2_, *θ*_2_) represent spherical coordinates for a coordinate transformation from the individual IMU frames to the knee joint frame, and *k* indicates the discrete time index as follows:

(3) $$t_{k}=k\Delta t$$

The minimization of the cost function in Equation (2) is established as a means of finding the best alignment of the two sensor frames to minimize the angular velocity along the knee axis. This equation is derived based on the assumptions that the knee joint can be approximated as a hinge joint and that most of the angular velocity should occur in the sagittal plane. Following this alignment, the knee flexion angle can be calculated using gyroscopes through integration as follows:

(4) $$\alpha_{gyr}\left(t\right)=\int_{o}^{t}\left(g_{1}\left(\tau \right)\cdot j_{1}-g_{2}\left(\tau \right)\cdot j_{2}\right)d\tau$$

After the joint axes have been determined, the coordinates of the joint centers in the local sensor coordinates, *o*_1_ and *o*_2_, are determined from an additional optimization procedure as follows:

(5) $$\begin{array}{l} \tilde{\Psi }\left({\hat{o}}_{1},{\hat{o}}_{2}\right)=\sum_{k=1}^{N}{\left[{\left\|a_{1}\left(t_{k}\right)-\Gamma_{g_{1}\left(t_{k}\right)}\left({\hat{o}}_{1}\right)\right\|}_{2}-{\left\|a_{2}\left(t_{k}\right)-\Gamma_{g_{2}\left(t_{k}\right)}\left({\hat{o}}_{2}\right)\right\|}_{2}\right]}^{2}\\ \Gamma_{g\left(t\right)}\left(\hat{o}\right)=g\left(t\right)\times \left(g\left(t\right)\times \hat{o}\right)+\dot{g}\left(t\right)\times \hat{o}\\ \begin{array}{cc} o_{1}={\hat{o}}_{1}-j_{1}\frac{{\hat{o}}_{1}\cdot j_{1}+{\hat{o}}_{2}\cdot j_{2}}{2}, & o_{2}={\hat{o}}_{2}-j_{2}\frac{{\hat{o}}_{1}\cdot j_{1}+{\hat{o}}_{2}\cdot j_{2}}{2} \end{array} \end{array}$$

The optimization algorithm presented in Equation (5) consists of correcting the acceleration signals for the normal and tangential acceleration components due to the position of the sensors relative to the joint. This type of correction is sometimes referred to as a lever arm correction. These joint centers are then used to correct the accelerometer measurements as follows:

(6) $${\tilde{a}}_{i}\left(t\right)=a_{i}\left(t\right)-\Gamma_{g_{i}\left(t\right)}\left(o_{i}\right),\;\;\;i=1,2$$

which are then used to identify the joint angle using the accelerometer data as follows:

(7) $$\begin{array}{l} \alpha_{acc}\left(t\right)={∢}_{2D}\left(\left[\begin{array}{c} {\tilde{a}}_{1}\left(t\right)\cdot x_{1}\\ {\tilde{a}}_{1}\left(t\right)\cdot y_{1} \end{array}\right],\left[\begin{array}{c} {\tilde{a}}_{2}\left(t\right)\cdot x_{2}\\ {\tilde{a}}_{2}\left(t\right)\cdot y_{2} \end{array}\right]\right)\\ \begin{array}{cccc} x_{1}=j_{1}\times c, & y_{1}=j_{1}\times x_{1}, & x_{2}=j_{2}\times c, & y_{2}=j_{2}\times x_{2}, \end{array} \end{array}$$

where ${∢}_{2D}\left(\cdot \right)$ denotes the signed angle between two vectors in $R^{2}$, and *c* is any vector not parallel to *j*_1_ or *j*_2_. For this study, we use $c={\left[\begin{array}{ccc} 1 & 0 & 0 \end{array}\right]}^{T}$. The coordinates (*x*_1_, *y*_1_) and (*x*_2_, *y*_2_) represent arbitrary 2D coordinates in the knee joint plane and are used as a means for determining the angle between the two IMU sensors.

The joint angle estimates from Equations (4) and (7) can then be combined using sensor fusion to get a better overall estimate. This is a necessary step because the gyroscope estimate from Equation (4) is prone to drift over time owing to integration, while the accelerometer estimate from Equation (7) exhibits noise and is less reliable at moments of large acceleration changes [17].

#### 2.2.2. IMU Data Processing and Modifications to the Seel et al. Algorithm

The overall general procedure outlined in Section 2.2.1 is implemented in this study. However, the information flow through this methodology is modified strategically in this study, specifically for the application of running. This is motivated by the significant errors encountered in the accelerometer knee flexion angle estimates from Equation (7) when implementing the methods from Section 2.2.1 directly. Owing to the high-speed conditions during running, the dynamics of the leg-mounted sensors are subjected to significantly higher accelerations than the walking data considered in Seel et al. [17]. Additionally, the higher impact due to foot strikes results in large acceleration spikes in the measurement data. However, because these peaks tend to be clearly identifiable within the accelerometer data, these accelerometer spikes are used to our advantage to detect foot strikes using a simple peak detection algorithm [44]. Note that the peak detection algorithm is applied to unfiltered accelerometer data. Then, the data between the foot strikes from 40% to 80% of each gait cycle are used for the gyroscope optimization procedure, as outlined in Equation (2). These portions of the gait cycle give a better alignment and identification of the joint plane based on the dynamic conditions typically encountered during those phases of the gait cycle. In other words, this specific selection of data offers relevant knee angle dynamics while reducing other effects on the motion, such as soft-tissue artifacts. Additionally, this reduces the other effects of the observed dynamics due to the impact and ground reaction.

After the joint axes in the local sensor coordinate frames, i.e., *j*_1_ and *j*_2_, have been determined, the accelerometer and gyroscope signals are filtered using a 4th-order zero-lag low-pass Butterworth filter with a 7 Hz cutoff frequency [20]. The filtered accelerometer signals are used to determine the projected angular velocity components as follows:

(8) $$\begin{array}{cc} {\tilde{g}}_{i}\left(t\right)=g_{i}\left(t\right)\cdot j_{i}, & i=1,2 \end{array}$$

The filtered accelerometer data corresponding to 40%–80% of each gait cycle are then sent to the optimization algorithm in Equation (5) to determine the joint centers, *o*_1_ and *o*_2_, which are then used to determine the corrected accelerometer signals using Equation (6).

**Figure 3:**
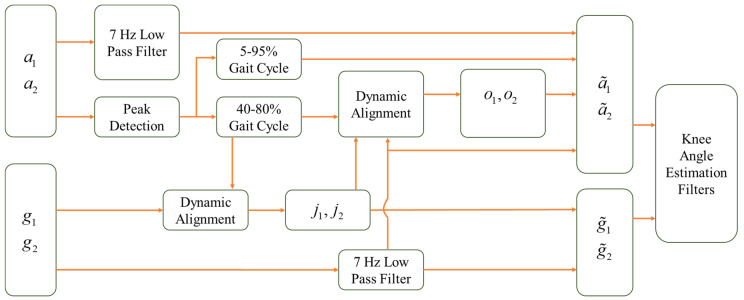
Flowchart summarizing the data processing prior to the knee flexion angle estimation filtering algorithms.

In addition to strategically selecting a certain portion of the gait cycle for the optimization portions of the algorithm, one additional modification is considered. That is, owing to the significant effects of impact and soft-tissue artifacts on the accelerometer signals around the impact, a certain portion of the accelerometer data is omitted from the measurement update of all the considered filtering algorithms. Specifically, the accelerometer data corresponding to 5% of the gait cycle before and after each detected accelerometer peak are not used for the measurement update portion of any considered filtering algorithm. Note that during this time, each filtering algorithm relies on gyroscope-only estimation for the knee flexion angle, which is reliable for short time periods. This is a reasonable assumption for the running application. A flowchart detailing the flow of information for the considered algorithm is provided in Figure 3.

### 2.3. Considered Filtering Algorithms

#### 2.3.1. Kalman Filter (KF)

Consider a linear discrete-time state space system given by

(9) $$\begin{array}{l} x_{k}=F_{k-1}x_{k-1}+G_{k-1}u_{k-1}+w_{k-1}\\ y_{k}=H_{k}x_{k}+v_{k} \end{array}$$

where *x* is the state vector, *u* is the input vector, *y* is the output vector, and *w* and *v* are zero-mean Gaussian process and measurement noise terms, respectively. With these definitions, the linear KF algorithm can be summarized as follows. First, the mean and covariance, *P*, for the state are predicted using the following equations:

(10) $$\begin{array}{l} {\hat{x}}_{k|k-1}=F_{k-1}{\hat{x}}_{k-1}+G_{k-1}u_{k-1}\\ P_{k|k-1}=F_{k-1}P_{k-1}F_{k-1}^{T}+Q_{k-1} \end{array}$$

where *Q* is the process noise covariance matrix. Then, the Kalman gain matrix, *K*, is calculated using the following equation:

(11) $$K_{k}=P_{k|k-1}H_{k}{\left(H_{k}P_{k|k-1}H_{k}^{T}+R_{k}\right)}^{-1}$$

where *R* is the measurement noise covariance matrix. Using this Kalman gain, the state and covariance estimates are updated with

(12) $$\begin{array}{l} {\hat{x}}_{k}={\hat{x}}_{k|k-1}+K_{k}\left(z_{k}-H_{k}{\hat{x}}_{k|k-1}\right)\\ P_{k}=\left(I-K_{k}H_{k}\right)P_{k|k-1} \end{array}$$

where *z* is the measurement of the output, *y*, and *I* is an identity matrix possessing appropriate dimensions.

#### 2.3.2. Extended Kalman Filter (EKF)

The EKF is an extension of the KF that works for nonlinear systems of the following form:

(13) $$\begin{array}{l} x_{k}=f\left(x_{k-1},u_{k-1}\right)+w_{k-1}\\ y_{k}=h\left(x_{k}\right)+v_{k} \end{array}$$

where *f* is the nonlinear state prediction function, and *h* is the nonlinear observation function. The EKF uses Jacobian matrices to handle the nonlinearity as follows:

(14) $$\begin{array}{cc} F_{k-1}={\left.\frac{\partial f}{\partial x}\right|}_{{\hat{x}}_{k-1},u_{k-1}}, & H_{k}={\left.\frac{\partial h}{\partial x}\right|}_{{\hat{x}}_{k|k-1}} \end{array}$$

where *F* is the state transition matrix, and *H* is the observation matrix. The EKF equations then follow a format similar to that of the KF and are summarized as follows:

(15) $$\begin{array}{l} {\hat{x}}_{k|k-1}=f\left({\hat{x}}_{k-1},u_{k-1}\right)\\ P_{k|k-1}=F_{k-1}P_{k-1}F_{k-1}^{T}+Q_{k-1}\\ K_{k}=P_{k|k-1}H_{k}{\left(H_{k}P_{k|k-1}H_{k}^{T}+R_{k}\right)}^{-1}\\ {\hat{x}}_{k}={\hat{x}}_{k|k-1}+K_{k}\left(z_{k}-h\left({\hat{x}}_{k|k-1}\right)\right)\\ P_{k}=\left(I-K_{k}H_{k}\right)P_{k|k-1} \end{array}$$

Note that the nonlinear functions *f* and *h* are used in the state estimate equations, while the Jacobian matrices are used for the covariance calculations like the KF.

#### 2.3.3. Complementary Filter (CF)

A CF typically consists of combining high-frequency information from one source with low-frequency information from another source. For state *x*, the CF algorithm can be summarized in the Laplace domain by [45]

(16) $${\hat{x}}_{k}=\frac{s}{s^{2}+\zeta_{CF}\omega_{CF}s+\omega_{CF}^{2}}\dot{x}+\frac{\zeta_{CF}\omega_{CF}s+\omega_{CF}^{2}}{s^{2}+\zeta_{CF}\omega_{CF}s+\omega_{CF}^{2}}x$$

where *ζ*_CF_ and *ω*_CF_ represent the damping ratio and natural frequency for the CF. Alternatively, specifically for the knee flexion angle application, an example CF for discrete time is offered in Seel et al. as follows [17]:

(17) $$\alpha_{acc+gyr}\left(t\right)=\lambda \alpha_{acc}\left(t\right)+\left(1-\lambda \right)\left(\alpha_{acc+gyr}\left(t-\Delta t\right)+\alpha_{gyr}\left(t\right)-\alpha_{gyr}\left(t-\Delta t\right)\right)$$

where *λ* is the fixed gain for the CF, which ranges from 0 to 1.

### 2.4. Knee Flexion Angle Estimation Algorithms

#### 2.4.1. Knee Flexion Angle Estimation using Kalman Filter (KF)

The KF algorithm considers the following state space definitions:

(18) $$\begin{array}{l} x\left(t\right)=\alpha \left(t\right)\\ u\left(t\right)={\tilde{g}}_{1}\left(t\right)-{\tilde{g}}_{2}\left(t\right)\\ y\left(t\right)=\alpha \left(t\right) \end{array}$$

With these definitions, the state dynamics are given in continuous time by

(19) $$\dot{x}\left(t\right)=\dot{\alpha }\left(t\right)=u\left(t\right)={\tilde{g}}_{1}\left(t\right)-{\tilde{g}}_{2}\left(t\right)$$

A first-order discretization is used to express the state dynamics in discrete time as follows:

(20) $$x_{k}=x_{k-1}+\Delta tu_{k-1}$$

which corresponds to a state space system of the form in Equation (9) with the following matrix definitions:

(21) $$\begin{array}{l} F_{k-1}=1\\ G_{k-1}=\Delta t\\ H_{k}=1 \end{array}$$

The output is a direct measurement of the state, which is the accelerometer-based estimate of the knee flexion angle, as provided by Equation (7), while the gyroscope information is used as an input to the filter. Effectively, the *Q* matrix, which is a scalar in this case, represents the uncertainty in the gyroscope measurement, while the *R* matrix, which is also a scalar in this case, represents the uncertainty in the accelerometer knee flexion angle estimate.

#### 2.4.2. Knee Flexion Angle Estimation using Extended Kalman Filter (EKF)

The EKF algorithm considers the following state space definitions:

(22) $$\begin{array}{l} x\left(t\right)=\alpha \left(t\right)\\ u\left(t\right)={\tilde{g}}_{1}\left(t\right)-{\tilde{g}}_{2}\left(t\right)\\ y\left(t\right)=\left[\begin{array}{c} {\tilde{a}}_{2}\left(t\right)\cdot x_{2}\\ {\tilde{a}}_{2}\left(t\right)\cdot y_{2} \end{array}\right] \end{array}$$

The coordinates (*x*_1_, *y*_1_) and (*x*_2_, *y*_2_) are determined from Equation (7). Note that owing to the selections of the state and input, the state prediction equation is linear in this case, resulting in the Jacobian matrix *F* = 1 for all time, and the discrete-time state prediction equation is the same as the KF, as in Equation (20). The output, however, is based on Equation (7) and is expressed in terms of a rotation from the shank sensor to the thigh sensor in the joint plane as follows:

(23) $$\left[\begin{array}{c} {\tilde{a}}_{2}\left(t\right)\cdot x_{2}\\ {\tilde{a}}_{2}\left(t\right)\cdot y_{2} \end{array}\right]=\left[\begin{array}{cc} \cos x\left(t\right) & -\sin x\left(t\right)\\ \sin x\left(t\right) & \cos x\left(t\right) \end{array}\right]\left[\begin{array}{c} {\tilde{a}}_{1}\left(t\right)\cdot x_{1}\\ {\tilde{a}}_{1}\left(t\right)\cdot y_{1} \end{array}\right]$$

This results in a nonlinear observation equation, which gives the following Jacobian matrix:

(24) $$H_{k}=\left[\begin{array}{c} -\sin x\left(t\right)\left({\tilde{a}}_{1}\left(t\right)\cdot x_{1}\right)-\cos x\left(t\right)\left({\tilde{a}}_{1}\left(t\right)\cdot y_{1}\right)\\ \cos x\left(t\right)\left({\tilde{a}}_{1}\left(t\right)\cdot x_{1}\right)-\sin x\left(t\right)\left({\tilde{a}}_{1}\left(t\right)\cdot y_{1}\right) \end{array}\right]$$

For the EKF, the *Q* matrix, like in the KF, is a scalar that represents the uncertainty in the gyroscope measurement. Here, however, the *R* matrix is a 2 × 2 matrix. A diagonal R matrix is assumed, with the first component corresponding to the uncertainty in the *x*-components of the acceleration in the joint plane and the second component corresponding to the uncertainty in the *y*-components of the acceleration in the joint plane.

#### 2.4.3. Knee Flexion Angle Estimation using Complementary Filter (CF)

The CF algorithm implemented herein is the same as the one offered in Seel et al. [17], as provided in Equation (17). Herein, the selection of the *λ* parameter represents the blend between the accelerometer- and gyroscope-estimated knee flexion angles. A higher *λ* parameter more heavily weights the accelerometer-based knee flexion angle estimates, while a lower *λ* favors the gyroscope-based estimates.

## 3. Results

First, a pilot dataset was collected from a 38-year-old female runner. The runner was a forefoot striker and ran an average of 12 miles per week. The sagittal plane knee angle ranged from 13.7° to 121.2° of knee flexion. The participant ran on a treadmill at 2.9 m/s, and running data were collected for 60 s following a 2 min warmup. These pilot data were used for developing and tuning the algorithm but not used for validation.

An additional series of data was collected from a different female runner, age 21, who was a rearfoot striker and ran an average of 14 miles per week. These data were used as validation data to evaluate the effectiveness of this proposed knee flexion estimation approach. Four different sets of data were collected for this participant. At the beginning of each dataset, the runner held a static T-pose for approximately 10 s. Then, the treadmill was set at a different speed, and the participant ran for approximately 3–4 min. The details for the treadmill settings are provided in Table 1.

### 3.1. Data Processing and Filtering

**Figure 4:**
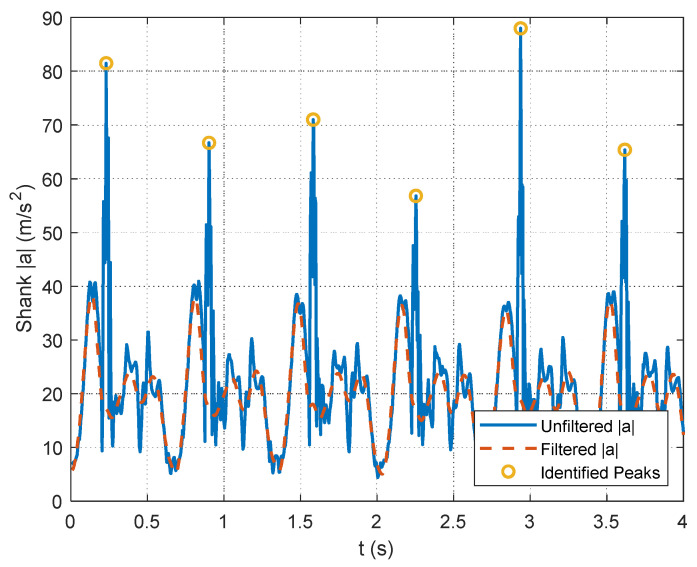
Illustration of peak detection used to identify foot strikes in the shank acceleration signal in the pilot data. The unfiltered magnitude of the shank acceleration is used to identify peaks, while the filtered acceleration signals are used within the estimation algorithms.

**Figure 5:**
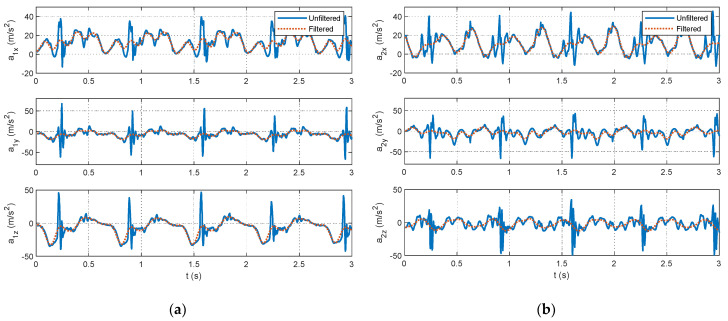
Excerpts from accelerometer measurements from the pilot data showing the unfiltered (solid line) and filtered (dotted line) accelerometer measurements from the pilot dataset for the IMU sensors mounted on the (**a**) shank and (**b**) thigh.

**Figure 6:**
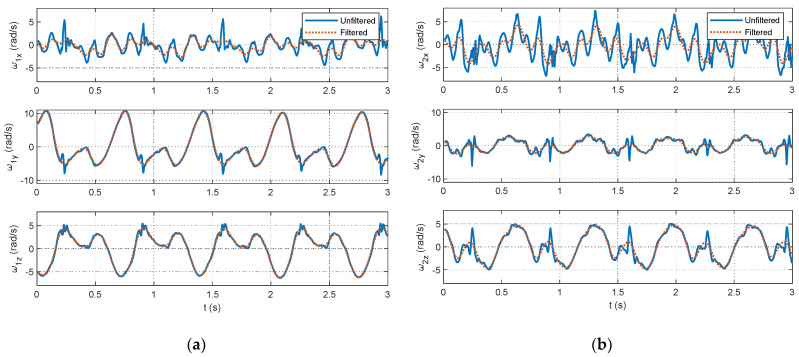
Excerpts from gyroscope measurements from the pilot data showing the unfiltered (solid line) and filtered (dotted line) gyroscope measurements from the pilot dataset for the IMU sensors mounted on the (**a**) shank and (**b**) thigh.

**Figure 7:**
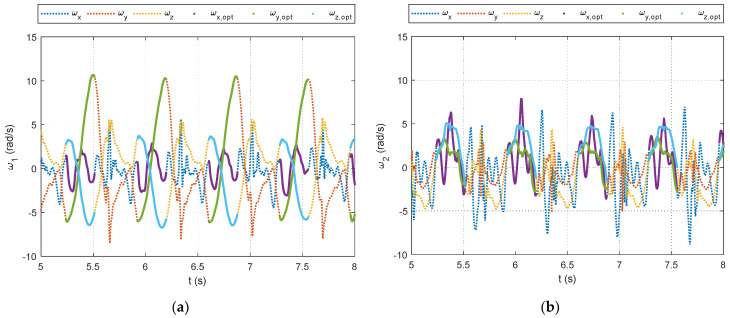
Excerpts from gyroscope measurements from the pilot data showing the original data (as dotted lines) and the regions that were selected for optimization (as solid lines) from the pilot dataset. (**a**) A portion of the shank gyroscope measurements. (**b**) A portion of the thigh gyroscope measurements.

**Figure 8:**
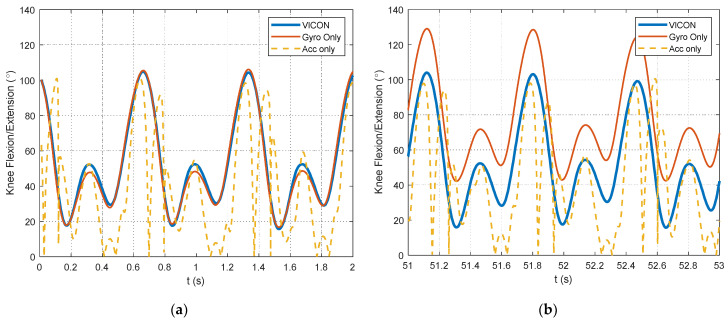
Excerpts from knee flexion angle estimation from gyroscope only and accelerometer only with respect to the Vicon motion-capture system reference measurements. (**a**) A portion of the knee flexion angle estimates at the beginning of the pilot dataset. (**b**) A portion of the knee flexion angle estimates toward the end of the pilot dataset.

**Figure 9:**
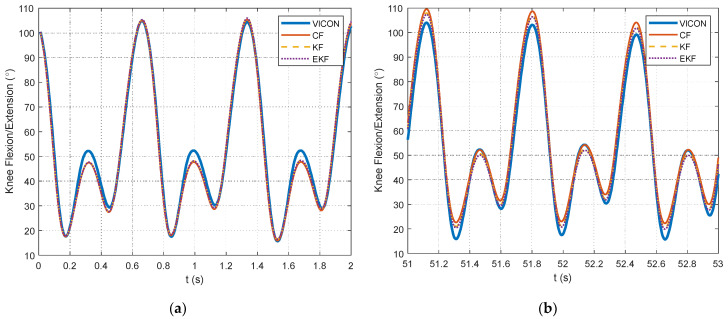
Excerpts from knee flexion angle estimations from CF, KF, and EKF with respect to the Vicon motion-capture system reference measurements. (**a**) A portion of the knee flexion angle estimates at the beginning of the pilot dataset. (**b**) A portion of the knee flexion angle estimates toward the end of the pilot dataset.

As outlined in Section 2.2.2, data processing is a key contribution of this study. The selection of data segments for the optimization relies on identifying foot strikes through a peak detection algorithm in the unfiltered accelerometer signals. The example figures in this section are provided from the pilot dataset. A plot showing the results of the peak detection is offered for a segment of pilot data in Figure 4. The large acceleration spikes due to foot strikes are distinguishable from the rest of the dataset and are marked with circles in Figure 4. After the peak detection is completed, the IMU signals are filtered. Example results for the 4th-order, zero-lag, low-pass Butterworth filtering at a 7 Hz cutoff frequency for the pilot data are shown for the accelerometers in Figure 5 and gyroscopes in Figure 6. Figure 5 and Figure 6 show the attenuation of the sensor noise as well as a reduction in the oscillations due to the foot strikes. Then, by leveraging the locations of the foot strikes, a portion of the gait cycle is selected for use in the optimization algorithms. An example plot illustrating this data selection for the pilot data is provided in Figure 7. In Figure 7, the segments of interest corresponding to the selected gait cycle percentages are identified and show the relevant dynamic behavior of interest for accurately identifying the joint plane. The results from gyroscope-only knee flexion angle estimates from Equation (4) and accelerometer-only knee flexion angle estimates from Equation (7) are shown as an example for the pilot data in Figure 8. Note that in Figure 8a, which shows data at the beginning of the dataset, the gyroscope estimates are close to the Vicon reference data while in Figure 8b, the gyroscope estimate shows significant drift, as expected, over time. However, the accelerometer estimate is generally not close to the Vicon reference data but does not exhibit any drift error. These results motivate the need for a well-tuned sensor fusion algorithm to combine the strengths of each individual sensor estimation. Example plots from the filtering algorithms for the pilot data are shown in Figure 9. Figure 9 shows reasonable agreement between the estimated signals and the Vicon reference data, thus demonstrating the feasibility of the algorithm. Each of the filters in Figure 9 show similar estimates, and the algorithms have the most significant errors in identifying some of the peaks of the knee flexion angle.

### 3.2. Comparison of Different Filtering Approaches for Knee Angle Estimation

**Figure 10:**
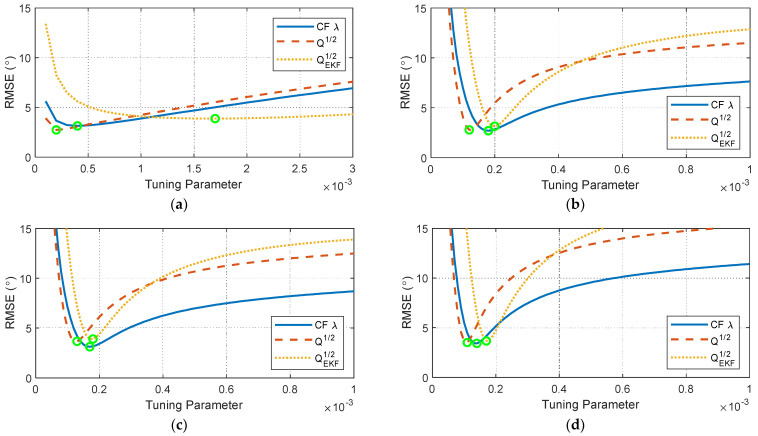
RMSE tuning results for the filter tuning parameters for (**a**) dataset #1, (**b**) dataset #2, (**c**) dataset #3, and (**d**) dataset #4.

The methods described in Section 2.2.1 and Section 2.2.2 were implemented for the four datasets outlined in Table 1. For this study, the root-mean-square error (RMSE) was selected as the metric to assess the accuracy and reliability of the estimation algorithms. For each filter, the parameters were manually tuned to minimize the RMSE of the estimated knee flexion angle with respect to the reference measurement obtained from the Vicon motion-capture system. This tuning procedure is illustrated in Figure 10 for each dataset. For the KF and EKF, the measurement noise covariance, R, was set at 1, and the process noise covariance was varied to adjust the blending between the gyroscope and accelerometer information. The lower values of the tuning parameters in Figure 10 correspond to the use of more gyroscope information relative to the accelerometer information, while the higher values of the tuning parameters indicate the use of more accelerometer information.

**Figure 11:**
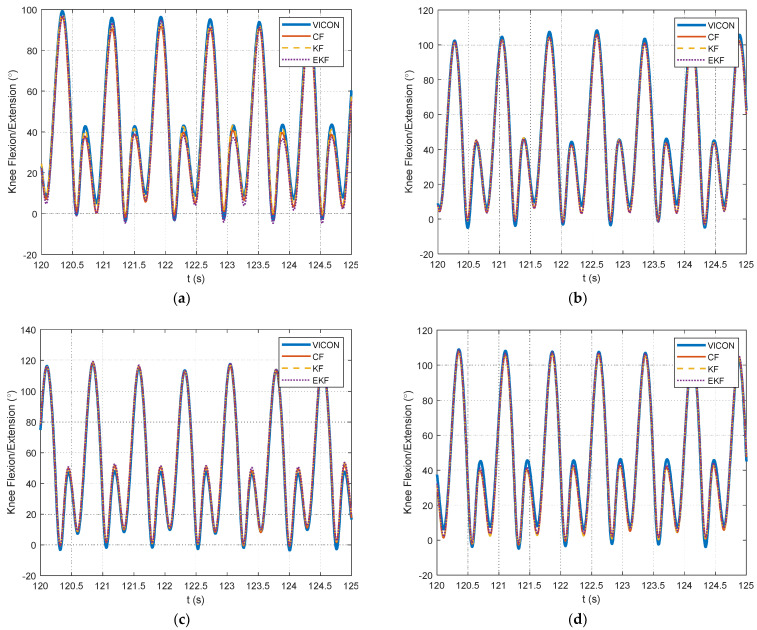
Illustration of knee flexion angles from CF, KF, and EKF with respect to the Vicon motion-capture system reference measurements from the validation data in (**a**) dataset #1, (**b**) dataset #2, (**c**) dataset #3, and (**d**) dataset #4.

**Figure 12:**
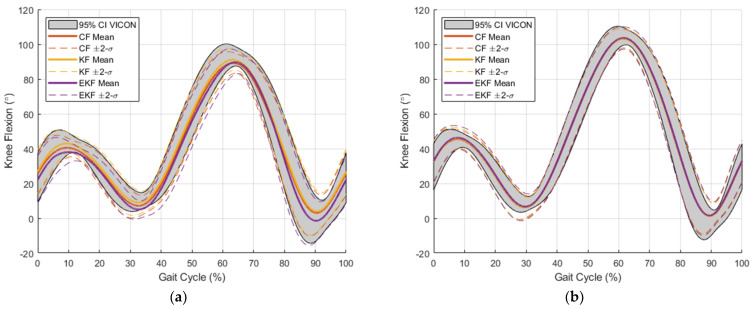

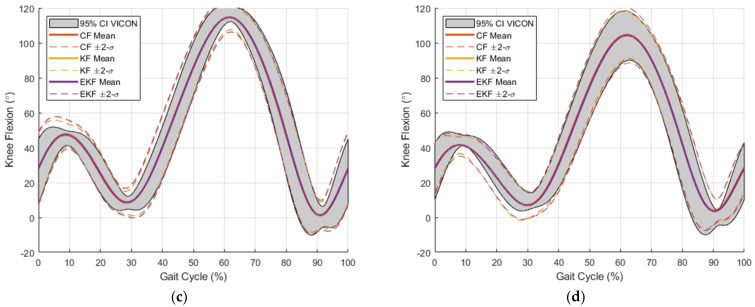
Visualization of knee flexion angle distributions from CF, KF, and EKF with respect to the Vicon motion-capture system reference measurements from the validation data in (**a**) dataset #1, (**b**) dataset #2, (**c**) dataset #3, and (**d**) dataset #4. The gray shaded region represents a 95% confidence interval for the motion-capture knee angle estimates. This represents the variation in the knee angle throughout the dataset. Mean (solid lines) and mean ± 2 standard deviations (dotted lines) are shown for each estimation filter.

Example plots of the knee flexion angle estimates are shown with respect to the motion-capture reference data for a portion of each validation dataset in Figure 11. For each filtering algorithm, the RMSE was calculated for the filters described in Section 2.4. The resulting RMSE values for each filtering algorithm are provided in Table 2. As an additional visualization of the knee angle estimations throughout the entire dataset, Figure 12 provides the mean and standard deviation for each gait cycle throughout the dataset. This information is shown along with a 95% confidence interval for the Vicon motion-capture data, which show the natural variation in the knee angle for the different steps taken throughout the dataset. It is shown in Figure 11 and Figure 12 that the range of the knee angle increases as the treadmill speed increases. Larger variations in the knee angle are shown in Figure 12 for dataset #4 relative to the other datasets. This is likely due to the varying treadmill speeds for this particular dataset. Also in Table 2 are the RMSE values for the knee flexion angle, using only the accelerometer measurement from Equation (7). This is provided as a benchmark to indicate how much the filtering algorithms can improve upon this accelerometer information by fusing it with the gyroscope information. Gyroscope-only estimates were not included owing to the significant drift over time that occurs when this information is unregulated. Using the same filter parameters as in Table 2, Pearson correlation coefficients were calculated for each of the filtering algorithm knee flexion angle estimates and are shown in Table 3.

## 4. Discussion

The results in Table 2 show a similar performance between the KF and CF algorithms, resulting in the best knee flexion angle estimates. The EKF algorithm yielded values close to those of the KF and CF but generally performed worse and, therefore, this particular implementation of the EKF is not recommended for this application. Owing to the mathematical structure of the EKF formulation, the filter effectively seeks to align the acceleration signals from the shank and thigh IMUs through a single rotation angle. Although the lever arm correction was applied to correct for the normal and tangential acceleration components relative to the knee joint, there were still potential differences in the acceleration signals due to the sensor noise, soft-tissue artifacts, etc., which likely led to the higher uncertainty for this formulation. Note that although the EKF, in general, as a filtering structure is more capable for handling certain aspects of the estimation problem than the CF owing to its time-varying gain calculation, in this instance, the underlying state space structure that was used to implement the EKF was inherently different from that in the CF formulation, thus resulting in the significant differences in performance.

In general, the accuracy of the knee angle estimates decreases as the treadmill speed increases, as shown in Table 2 for datasets 1–3. The faster running speeds result in more dynamic conditions, resulting in faster accelerations as well as more significant foot-strike impacts, which likely cause an increase in the error. Because the CF offered the lowest overall RMSE and was more computationally efficient than the KF algorithm, the CF method is recommended for this application, though the KF is a feasible approach. All three filters indicated a strong correlation with the motion-capture data, as shown in Table 3.

When comparing the filtering methods for the accelerometer-only data in Table 2, the gyroscope information significantly improves the knee flexion angle estimates. It is worth noting that in this application for running, the accelerometer-only estimates are quite poor. This is also apparent in Figure 8. This poor accelerometer estimate of the knee flexion angle significantly motivates this study. The walking dataset analyzed in Seel et al. [17] contained much lower accelerations and yielded much more accurate knee flexion angle estimates from the accelerometers. However, despite the significantly worse accelerometer performance in this application, a comparable amount of error was achieved in Seel et al. [17], which reported 3° of error for the human leg. Other researchers have reported RMSE errors for running speeds of approximately 5 mph of 3.4° [22] and 7.3° [21]. Jakob et al. also reported an RMSE error of 10.2° [21] for a speed of 3.0 m/s, which is close to the speed for dataset #2 in this paper. The proposed method in this study offers better results when comparing these values with the RMSE values from Table 2. The estimates shown in Table 2 satisfy the recommendation from McGinley et al. [46], which concluded that errors greater than 5° should not be used for gait analysis decision making. Therefore, the methods proposed in this study can be used as a portable and low-cost means for estimating knee angles for studying running gait kinematics. This has potential use in analyzing running in various outdoor applications.

The work in this study has some limitations. The results were obtained using a single set of pilot data and validated with respect to datasets from a single test participant. A more thorough dataset containing data from multiple human participants would be necessary to ensure the results are more generalizable. There may be differences in the accuracy of the algorithms owing to how the IMU sensors were attached to each participant. Another limitation of this study is that the filtering algorithms were initialized using the initial knee flexion angle, as determined by the motion-capture system. Ideally, this initialization would be obtained using only the IMU sensors or through other simple means, such as a goniometer. For practical implementation purposes, static IMU data, such as a standing pose at the start of the data collection, could reasonably be used to identify the initial condition. In this study, by initializing with the motion-capture system data, the results focused on analyzing the long-term sensor estimation errors rather than compounding these errors with additional uncertainty due to the initialization error. Future work should explore this initialization in more detail. Finally, the filter parameters were tuned manually for each dataset according to the calculated RMSE values. In practice, if this method is applied to the data without the motion-capture reference data, this type of tuning procedure would not be possible, so a more generally applicable tuning procedure is desirable. In real-world scenarios where the motion-capture reference data are not available, one possible approach for tuning would be to adjust the filter parameters until the gyroscope drift is sufficiently corrected. That is, when the tuning parameters are too low, a drift in the knee angle estimates is observed over time. This is readily apparent when viewing the peaks in the estimated knee signals, for example, as shown in Figure 8b. Incrementally increasing the tuning parameters until this drift becomes stabilized should result in a reasonable knee flexion angle estimate. If the parameter is increased too far, the estimation results will begin to favor the accelerometer angle estimates. This approach could be automated in future work, such as by minimizing the line fitted to the estimated knee flexion angle peaks.

This study was performed on a treadmill, which has known differences in knee kinematics compared with those of overground running, notably, a 2° increase in knee flexion at the foot strikes and a 6° increase in the knee flexion excursion on the treadmill as compared with those on a variety of overground surfaces [47]. This small kinematic difference is not expected to affect the method’s generalizable applicability to overground running, as it was validated using two runners with different overall knee kinematics that exceeded the differences expected on overground surfaces. However, it should be considered that the environmental considerations of outdoor, overground running may contain turns that may adversely influence the IMU readings. Future work should consider the transfer of this method to outdoor running over varied terrains.

## 5. Conclusions

This study presented a novel approach for knee flexion angle estimation using only shank- and thigh-mounted IMUs. The considered approach used strategically selected portions of the gait cycle through foot strike detection to help guide an optimization technique for aligning the sensors to the sagittal plane. This approach was evaluated using three filter variations for four sets of running data measured on a treadmill and validated with respect to reference data from a motion-capture system. The results of this study indicate that a complementary filter approach is the most effective for this methodology of knee flexion angle estimation for running applications.

## Figures and Tables

**Table 1:** Description of treadmill speeds for the test participant. The first three datasets used a fixed treadmill speed, while the fourth dataset contained varying speed conditions throughout the testing.

Dataset #	Speed Condition	Treadmill Speed (mph)	Treadmill Speed (m/s)
1	Constant	5.0	2.24
2	Constant	6.5	2.91
3	Constant	8.0	3.58
4	Varying	5.0–8.0	2.24–3.58

**Table 2:** RMSE values for each of the considered filtering algorithms in degrees for the proposed algorithm. The filter parameters were manually tuned to optimize the RMSE estimation results. RMSE values were calculated with respect to the Vicon and Visual3D reference measurements for the validation data.

Dataset #	Filtering Algorithm RMSE (°)			
	KF	EKF	CF	Accelerometer Only
1	2.726	3.876	3.140	23.09
2	2.758	3.148	2.703	27.69
3	3.636	3.866	3.110	28.61
4	3.460	3.639	3.415	31.90

**Table 3:** Pearson correlation coefficients for each of the considered filtering algorithms with respect to the Vicon and Visual3D reference measurements for the validation data.

Dataset #	Filtering Algorithm Correlation Coefficient			
	KF	EKF	CF	Accelerometer Only
1	0.9967	0.9978	0.9965	0.7735
2	0.9968	0.9958	0.9969	0.7424
3	0.9955	0.9948	0.9968	0.7729
4	0.9945	0.9934	0.9948	0.6776

## Data Availability

All the data that can be shared are included in the article. Not all the collected data can be shared owing to the privacy restrictions of the human subject research data, as required by the Institutional Review Board (IRB).
